# Testing the significance of pricing factors of oil and gas companies

**DOI:** 10.1371/journal.pone.0316147

**Published:** 2024-12-30

**Authors:** Antonio Garcia-Amate, Laura Molero-González, Miguel Angel Sánchez-Granero, Juan Evangelista Trinidad-Segovia, Andres García-Medina

**Affiliations:** 1 Department of Economics and Business, University of Almeria, Almeria, Spain; 2 Department of Mathematics, University of Almeria, Almeria, Spain; 3 Center for Research in Mathematics, Unidad Monterrey, Apodaca, Mexico; 4 Consejo Nacional de Ciencia y Tecnologıa, Mexico City, Mexico; Roma Tre University: Universita degli Studi Roma Tre, ITALY

## Abstract

For decades, fossil fuels have accounted for 70% to 80% of global primary energy demand. Far from ending this trend, O&G companies continue to be the main fore-runners in providing secure, versatile and widespread energy to the entire world. The relevance of this sector in the economic-financial landscape and the concern for its stability, makes that the high interest of the scientific community to explore the factors that explain the O&G cross-sectional expected returns remains intact. Through a new approach from the Random Matrix Theory, the aim is to know how many are the factors that explain the market performance of the O&G subsectors (upstream, midstream & downstream), and also if the Brent price can be considered an explanatory factor. We will show that for certain periods, Brent becomes the only factor explaining the movements in the upstream and midstream subsectors, while for most of the time the only factor is the market. Other interesting finding is that no significant factors are found for the downstream subsector, except in certain periods. With a purely statistical approach, we show the factors that explain the cross-sectional expected return of the O&G companies, providing information of special relevance for the decision making of investors, executives and politicians.

## 1. Introduction

For decades, fossil energy sources has accounted for 80% of global energy demand [1], a scenario that, far from coming to an end, will continue for a few more decades. Organizations such as the Organization of Petroleum Exporting Countries [2] and the Energy Information Administration [3] warn that by 2045–2050 these fossil energy sources will account for around 70% of global energy demand. It is observed how Oil and Gas (hereinafter, O&G) will continue to be protagonists of the global energy mix, despite the efforts in investment to transform the economy to a more sustainable and cleaner one [4]. This reality raises the need to understand and study how the O&G sector perform in financial markets in order to mitigate possible future shocks that could harm these companies [5, 6]. The high capitalization of this sector and the energy dependence on O&G makes this energy source play a key role in the global economy [7]. Therefore, researchers, politicians and investors have been attracted by the need to study the valuation of the O&G sector in financial markets and the factors that explain its performance [8, 9].

The goal of this research is to question the factors that explain the O&G stocks performance that have been promulgated for decades in research, financial markets, and economic outlook. Additionally, the research disseminates the O&G sector into upstream, midstream, and downstream subsectors with the intention of delving deeper into the possible factors that explain the performance of independent O&G companies [6]. In order to achieve this goal, the problem is studied from a novel approach based on Random Matrix Theory (RMT) models, particularly the Onatski test [10]. Due to the large sample used, the analysis is performed in the context of high dimensionality, thus allowing us to tackle the problem from a purely statistical point of view. Until now, the literature has focused on determining whether the proposed factors are or not statistically significant. With this methodology, the perspective from which the problem is addressed changes. We focus on determining who many factors are significant in explaining the cross-section of stocks returns [11].

The relationship between crude oil price and financial markets has been intensively studied over the past four decades [12–16]. Seminal articles such as Sadorsky [17] supported a negative relationship between oil prices and U.S. stock markets. The same author in 2001 introduced interest rates and foreign exchange rates as explanatory variables, outside the oil price [18]. The most widespread view is that oil price is a key factor affecting financial markets [19–23]. Through the Fama-French factors and oil futures return, Elyasiani et al. [24] found evidence that oil price is a systematic risk factor for nine of the thirteen industries analyzed, presenting statistical significance in the relationship between oil price and industry excess return. Others such as Reboredo & Rivera-Castro [25] find that for the financial crisis there is contagion effect between changes in oil price and return in financial markets. By contrast to the most of papers published in previous literature, some analyses disprove this significant relationship. Authors such as Huang et al. [14], Cong et al. [26] and Zhang [27] did not find a significant relationship between oil price and excess returns in financial markets. In particular, and due to the relevance of the O&G sector in the financial landscape, one also finds literature focused on studying the factors affecting this sector. For a sample of Canadian O&G companies, Boyer & Filion [28] support that stock returns are positively related to the price of crude oil. Others such as Mohanty & Nandha [29] and Adekunle et al. [30] present similar results for the O&G sector in the United States and Nigeria, respectively.

Although the valuation of O&G companies within financial markets has been intensively studied, to the best of our knowledge, there are a limited number of papers that differentiate this large sector by subsectors. The O&G chain encompasses heterogeneous activities, and should be studied separately to draw more objective conclusions [8, 29]. Recently, authors such as Lyócsa & Todorova [31] have indicated how little attention is paid to this differentiation and how important it is to study the O&G sector separately. In line with the distribution drawn from Kang et al. [32] and Ewing et al. [33], this paper studies the three subsectors covered by the O&G sector (upstream, midstream and downstream) thus allowing to cover this gap indicated in the literature.

Another debate within the valuation of the O&G sector is the methodology used. According to Sanusi & Ahmad [9], the most common methods to study the O&G stock returns-oil price link are cointegration analysis, multifactor regression and volatility spillover analysis. The theoretical framework for studying the O&G stock returns-oil price link is the risk-return dichotomy based on the Capital Asset Pricing Model (CAPM) [14, 15]. Others such as Hoque & Low [34] or Jouini [22] indicate that Arbitrage Pricing Theory (APT) models are more recommendable as they can explain stock performance through a larger number of factors. Far from consensus, there is a need to apply more sophisticated statistical methodology to the study of the O&G stock returns with a global perspective [35]. In the financial field, Random Matrices have been applied to the Markowitz model [36]. Through mathematical statistics, these techniques have evolved to allow objective signals to be extracted within the framework of Principal Component Analysis (PCA). These new techniques were promoted by Onatski [10], thus making it possible to go further, with respect to APT models.

Despite considerable debate, there remains no global consensus within the literature on the explanatory factors for the stock performance of O&G companies. This study employs a novel approach based on RMT to identify the number of factors that significantly impact the share price. By understanding these factors, shareholders and investors can make more informed investment decisions based on proven empirical evidences. The results highlight a distinct behaviour pattern along the O&G industry activity chain, thereby substantiating the necessity for a separate analysis of each O&G subsector.

The remainder of the paper is structured as follows: Section 2 reviews the most relevant literature; Section 3 presents the methodology employed; Section 4 describes the sample, the results and present the discussion; Conclusions and practical implications are given in Section 5.

## 2. Literature review

According to a recent study published by Energy Institute [37], O&G accounted for 55% of the primary energy consumed globally in 2022. This sector plays a key role in the global economic panorama, which makes it extremely important to know what are the factors that affect the value of the companies that belong to it [8, 38]. Due to its relevance and the volatility experienced by the sector in certain periods [39], the study of how many and what factors affect is fundamental for the decision-making of politicians, executives and investors in the financial markets. From the beginning, the oil price has been considered a significant factor affecting the excessive stock returns. The theoretical support for this relationship is found in the stock valuation model based on discounted cash flows [40, 41], which indicates that the oil price can affect the stock prices through two channels. First, the oil price, as one of the most consumed energy sources, affects the cost of commodities, which affects the cost of production and ultimately a reduction in net profit and stock value [42]. Second, a rise in the oil price can cause inflation to increase, and therefore central banks will consider raising interest rates. This will cause the cost of capital to rise, thus increasing the discount ratio and producing a decrease in the share values [43]. In the 90s, authors such as Kaneko & Lee [44] or Jones & Kaul [15] already documented this relationship empirically, supporting a significant impact of oil prices on the U.S, Canada, Japan and the United Kingdom financial markets. Saeed [45] indicate that the oil price has a significant impact not in all sectors, but in some closely related to crude oil such as the O&G, automotive or electronics sectors. Zhang [27] is in line with this stance, indicating that there is widespread consensus that oil shocks affect, but not all industries in the same way. Due to this heterogeneity in the impact of price, and considering that the O&G sector is a priori closely related, the scientific community began to show interest in valuation models applied to the energy industry. Manning [46] was one of the first to study the British O&G sector, relating the oil price return in the 80s with the O&G companies. It found that exploration O&G companies react more strongly to price increases than integrated O&G companies. Through the APT multifactorial model, Al-Mudhaf & Goodwin [47] analyzed the price of 29 US O&G companies and its relationship to the 1973 oil shock, finding evidence that the oil price increased the companies’ return. For the Australian O&G sector, Faff & Brailsford [19] used a two-factor model including market beta and oil price as a risk factor. They found that price was significantly related to the companies’ return. More recent authors such as Sanusi & Ahmad [9] or Ebechidi & Nduka [48] find significant relationship between the energy industry and factors such as market risk, size, factors related to book-to-market or exchange rates.

At the beginning of the twentieth century, authors such as Perry Sadorsky [18] attracted the attention of the research community to focus efforts on knowing what were the factors that affect O&G stock returns. For the Canadian O&G sector, the author found that the reference market and the oil price had a high impact on the stock return volatility. Then, the price became a source of risk for these companies. In line with these results and also focused on Canadian energy stocks, Boyer & Filion [28] found that the appreciation of crude oil was positively related to the stock price through the increase in the cash flows of companies. Shaharudin et al. [49] found for the main stock indices of the United States, London and India a significant relationship between the oil price and O&G companies, as well as a significant effect of other variables such as interest rates or the reference market index. However, authors such as El-Sharif et al. [50] find a positive relationship between the oil price and the British O&G sector, but not always highly significant. Chang et al. [51] present results where a very low correlation is observed between the future of the WTI price and the stocks of ten O&G companies. Others such as Mohanty et al. [52] found no significant relationship between the oil price and the stock prices for the period 1998–2010, indicating that no O&G company is significantly exposed to oil price risk.

These mixed results laid the groundwork for the debate on whether the O&G sector should be considered as a whole or differentiated by type of activity within the O&G chain. Following Swaray & Salisu [4], there is a “homogeneous expectation” on investors and fund managers that the return and risk of O&G companies that are at different levels of the value chain (upstream, midstream and downstream) perform in the same way and are affected by the same number of factors. It is extremely necessary to perform analyses at the company level, thus avoiding wrong results caused by an aggregate study or at the sector level [29]. For instance, in the case of downstream companies, they have the ability to pass high crude oil prices to final consumers through the increase in the derivates prices, thus reducing the possible negative impact of this increase [28]. According to a study conducted by Liu [7] where he differentiated his sample by subsectors, Exploration & Production (E&P) companies and Equipment & Services (E&S) companies, he found that the reaction to the oil price differed from one subsector to another. Akhtaruzzaman et al. [53] demonstrated for North and South American countries that the E&P subsector and integrated companies had greater exposure to oil price risk, compared to O&G infrastructure providers such as the E&S subsector or pipelines. A recent paper by Carson [54] demonstrates empirically for the period January 2000-August 2020 that the pipelines and Refining & Marketing (R&M) subsectors are not systematically related to crude oil price movements. On the other hand, due to the proximity to the commodity, the E&P and E&S subsectors do have a great price risk.

In summary, the valuation of the O&G sector should be studied according to specific activities due to: (i) differences in the probability and effect of hedging the risk of future prices [55]; (ii) how close the company is to oil extraction [29]; (iii) the ability of the subsector to pass on oil price increases to their consumers [28, 52, 56]; (iiii) the ability to manage inventory and absorb price volatility [57]. Another of the most relevant issues that explain the disparity of results is the methodology and the sample used. Previous studies often use benchmark stock indices to assess the O&G sector or the impact of oil prices on other sectors [27, 58–60]. However, it is well known that this can lead to certain deficiencies in the results extracted [61]. The location of the sample is another relevant factor to highlight, because according to Tsuji [35] one of the biggest challenges in the valuation of O&G is to tackle the apparent absence of a global perspective. Indeed, much of the previous literature focuses on a specific market: Canada [28], India [49], U.S [29], United Kingdom [9], Nigeria [30], Malaysia [34], among others. The use of high-frequency data (daily data) or the correct choice of the applied model are other pending tasks in this type of analysis [35]. Most studies related to O&G valuation use standard multifactor asset pricing models to carry out the analyses [34]. Carson [62] uses the Fama-French model of three-factors augmented (including the WTI price of crude oil and the Henry Hub of natural gas) to value the U.S O&G sector. Years later, the same author uses the Fama-French model again, this time the five-factor model [54]. These issues set the starting point for the present investigation.

In the field of Finance, modelling using factors is commonly employed for dimension reduction in time series analysis [63]. Authors as De Nard et al. [64] have introduced factor structure for estimating time-varying and high-dimensional covariance matrices.

Freyberger et al. [65] apply a lasso strategy in a non-parametric regression model under conditions of high-dimensionality. Bryzgalova et al. [66] propose a Bayesian estimator to determine the number of statistically significant factors in explaining the cross-section of stocks returns. Similarly, Kozak et al. [67] presented a Bayesian approach to determine de Stochastic Discount Factor (SDF) coefficients on a high-number of characteristic-based factors.

In this paper, we propose to deal with high-dimensionality through the Random Matrix Theory (RMT). The applications of this theory to Finance have been focused mainly on the analysis and cleaning of the covariance matrix in the Markowitz Mean-Variance model [36, 68]. These techniques were formalized within the field of mathematical statistics [69]. Statistical tests and estimators began to been proposed to determine the true signals in the context of Principal Component Analysis (PCA). The formalization of RMT within statistics make possible studies regarding more general financial models, as APT models. The authors Molero-Gonzalez et al. [11] began this line of study of the number of statistically significant factors for the S&P500 and Nasdaq markets, concluding that only the market factor resulted to be statistically significant. In this paper, we expand the study for the O&G industry, adding clusters by subsectors.

## 3. Methodology

The Random Matrix Theory (RMT) is presented as a new type of statistical mechanics where instead of having a group of stated governed by the same Hamiltonian, we have a set of Hamiltonians governed by the same symmetry. Wishart [70] was the first in introducing this theory into mathematical statistics. The theoretical foundations of RMT can be traced back to the 1950s decade. It was then that Wigner proposed a statistical description of the energy levels of the uranium nucleus [71] employing RMT. In 1962, Dyson extended the ideas of Wigner, showing that physically reasonable symmetry assumptions can be represented by Gaussian ensembles [72]. This theory gained momentum when Bohigas et al. [73] stated the quantum chaos conjecture. The connection between RMT and finance it is owed to Laloux et al. [74] and Plerou et al. [75]. With their works authors propose to use RMT to model the interactions of financial markets through the Wishart ensemble (a set of covariance matrices with Gaussian inputs distributed under the Haar measure). The general implications of this ensemble are frame within multivariate statistics.

When discussing high-dimensionality, it is well known that the covariance matrix is distributed according to the Wishart [70] distribution. Consider *X* a matrix of dimension *n* x *p*, with random entries i.i.d. according to a Gaussian probability distribution function (PDF), with zero mean and variance equal one. Each vector *X*_*i*_ of dimension *p* x 1 is denoted as *X*_*i*_ *~ N*_*p*_ *(0*, *Σ)*, *i* = 1, …, *n*. In statistics, **W** = **X**^T^**X** is said to have a p-variant Wishart distribution of *n* freedom degrees *W*_*p*_ *(n*, *Σ)*.

Within the context of RMT, it is said that the matrices with structure W belong to the Wishart Orthogonal Ensemble (WOE). These matrices are rotationally invariant under the Haar measure. A universal result is that, independently the particular matrix X, when the number of columns (*p*) is of the same order as the number of rows (*n*), and both dimensions grow with no limit, the distribution of the eigenvalues of matrix W converges to what is known as the Marchenko and Pastur [76] law:

$$\rho (\lambda )=\frac{\sqrt{\left(\lambda_{+}-\lambda \right)\left(\lambda -\lambda_{-}\right)}}{2\pi q\lambda },\lambda_{\pm }={\left(1\pm \sqrt{q}\right)}^{2}$$

where it is assumed that *Σ =* I.

If we focus in the financial context, *X* is the data matrix of returns. The first dimension represents the number of days and the second dimension the number of assets. *W* becomes the covariance matrix of *p* returns time series of length *n*. The Marchenko-Pastur law implies a bias in the estimation of the covariance matrix that increases as *q = p/n →∞*. Focusing on asset pricing, the problem relies on the factor models, which have the covariance matrix as their central object. Thus, it is necessary to consider the effect of dimensionality to avoid bias in estimating the number of factors that determine the price of stocks. To achieve this goal, the Tracy and Widom [77] distribution is considered. This distribution elucidates the behavior of the eigenvalues near the boundaries of the Marchenko-Pastur law. Concretely, this distribution helps to distinguish between the eigenvalues within the noise zone and those that are true signals. By boosting the Tracy-Widom distribution, hypothesis tests can be constructed to formally differentiate this bias.

In 2008, Onatski [78] extended this previously mentioned work and proved that the joint distribution function of the largest normalized centered eigenvalues of a complex p-dimensional Whisart matrix converges to the Tracy-Widom [77, 79] distribution, as *p*,*n*→∞, so that *p*⁄*n* = *q* ∈ (0,∞). The author demonstrates how the theoretical result of his work can be employed to find the set of common factors in stock returns, with a level of confidence of 95%. In 2009, Onatski [10] develops a null hypothesis test that there are *k*_0_ number of factors against the alternative hypothesis that there are more than *k*_0_ factors, but less than *k*_1_+1, in a generalised dynamic factor model (DFM). The test is based on a $\hat{R}$ statistic, proposed to determine the number of factors in the generalized DFM of Forni et al. [80]:

$$\hat{R}=\max_{k_{o}<i\leq k_{1}}\frac{{\hat{\lambda }}_{i}-{\hat{\lambda }}_{i+1}}{{\hat{\lambda }}_{i+1}-{\hat{\lambda }}_{i+2}}$$ (1)

where ${\hat{\lambda }}_{i}$ is the i-th largest eigenvalue of the smoothed periodogram estimate of the spectral density matrix of the data at a predetermined frequency. The author proved that the statistic of the test is asymptotically pivotal and has a distribution related to that of Tracy Widom type 2. Onatski tabulated the percentiles of the distribution, providing a set of critical values for the hypothesis test, which are shown in S1 Table online.

The model considers a total of *n* observations with the structure $X_{t}=\Lambda (L)F_{t}+e_{t}$; where Λ(*L*) is the polynomial matrix of size *p* × *n* in the delay operator *L*, *F*_*t*_ is a k-dimensional vector of factors at time *t*, and *e*_*t*_ is a p-dimensional vector of stationary correlated idiosyncratic terms.

A particular case occurs when Λ(*L*) does not depend on *L*, but factors do no necessarily follow a white (Gaussian) noise process. In this case, the dynamic factor model is reduced to the approximated factor model of Chamberlain & Rothschild [81] and the idiosyncratic terms are gaussian and independent over time, so that we can prove the hypothesis of the approximated number of factors (in contrast to the dynamic one).

The steps followed in this study to determine the number of statistically significant factors were as follows:

I. Given the data matrix *X*, it is divided into two periods of time of equal size and the complex matrix ${\hat{X}}_{j}$ is constructed:
$${\hat{X}}_{j}=X_{j}+iX_{j+\frac{n}{2}}$$ (2)
II. We, then, compute the eigenvalues ${\hat{\lambda }}_{i},\ldots ,{\hat{\lambda }}_{p}$ of
$$\frac{2}{n}\hat{X}{\hat{X}}^{*}$$ (3)
  where * denotes the transpose of the conjugate of the matrix.
III. Thirdly, the $\hat{R}$-statistic is calculated through expression 1.
IV. Finally, the null hypothesis *H*_0_ about the existence of *k*_0_ factors is accepted or rejected, according to the value of $\hat{R}$ and as indicated in the table of critical values provided by Onatski [78].

The basis of the test consists of the idea of the spectral separation and is related to the joint Tracy-Widom distribution for the Gaussian Orthogonal Ensemble (GUE) with *β* = 2 [68]. When *k* is the actual number of factors, the first *k* eigenvalues are separated from the bulk and fall off the scale. The argument is based on the fact that the data matrix $\hat{X}$ has a structure that is given by a factor model. The covariance matrix of the idiosyncratic errors is assumed to follow a Whisart distribution with complex entries. Then, given that $\hat{X}$ has a k-factor structure, the eigenvalues ${\hat{\lambda }}_{1},\ldots ,{\hat{\lambda }}_{k}$ diverge as *p*,*n*→∞; while ${\hat{\lambda }}_{k+1},\ldots ,{\hat{\lambda }}_{p}$ converge to the Marcenko-Pastur distribution with *β* = 2 [76], as they are complex entries. Thus, given that *λ*_*k*_→∞ and that ${\hat{\lambda }}_{k+1}$ and ${\hat{\lambda }}_{k+2}$ remain bounded by approximation to the Marcenko- Pastur distribution, we have that the $\hat{R}$-statistic diverges in the asymptotic regime *p*,*n* → ∞ [11].

In practical terms, the procedure to determine the number of factors consists on applying the Onatski’s test iteratively, testing on each iteration a different number of factors until the null hypothesis is reached. In this way, it is supposed, a priori, that the actual number of factors (*k*) takes a value comprised between *k*_1_ and *k*_2_. Subsequently, we test *H*_0_:*k* = *k*_1_ against *H*_1_:*k*_1_ < *k* ≤ *k*_2_, with a significance level of *α*. If *H*_0_ is accepted, it is concluded that the estimated number of factors is *k*_1_ and the algorithm stops. If, on the other hand, *H*_0_ is rejected, we test *H*_0_:*k* = *k*_1_+1 against *H*_1_:*k*_1_+1<*k* ≤ *k*_2_. This procedure is repeated until *H*_0_ is accepted. The corresponding number of factors is considered to be the best estimation of the associate model.

## 4. Empirical application

### 4.1 Data

In this study, companies belonging to the O&G sector were taken. Three datasets have been created; the sample has been divided into the three subsectors: companies belonging to the upstream subsector, companies belonging to the midstream subsector and companies belonging to the downstream subsector. A company selection criterion has been established, whereby only those companies in which the number of days with missing closing prices do not exceed 5% were taken and its logarithmic returns were estimated. In this way, *p* = 142 companies for the upstream dataset; *p* = 81 companies for the midstream dataset; and *p* = 24 companies for the downstream dataset. The study period considered starts on 1^*st*^ January 2005 and ends on 31^*st*^ December 2022.

In the context of random matrices, there’s a determining parameter, which is given by the ratio between the number of stocks (*p*) and the number of observations (*n*):*q* = *p*⁄*n*. Particularly, the noise effect due to the finiteness of the sample is observed for values of *q* → 1. In this study we have considered two values for *q*, in order to analyze the explanatory power of the test: *q* = 1/8 and *q* = 1/2. When considering *q* = 1/8, we talk about low dimensionality and it can be solved employing classical statistical techniques. We talk about high dimensionality when *q* = 1/2. This second scenario requires modern techniques, such as the Tracy-Widom and Onatski statistical test, which fall within the scope of Random Matrix Theory.

For the study, a sliding window experiment has been designed for each subsector and value of *q*. For each window, the number of statistically significant factors was determined at 3 different confidence levels *α* ∈{0.01,0.05,0.10}. We have considered an upper bound *k*_2_ = 8. In this way, the alternative hypothesis has been tested only up to that limit. We consider that this limit results to be enough to cover most situations.

The number of observations for the upstream stocks is *n* = 284 days, for *q* = 1/2, and *n* = 1136 days, for *q* = 1/8. For the midstream stocks, *n* = 162 days, for the high dimensionality scenario, and *n* = 648 days, for the low di- mensionality scenario. In the case of the downstream dataset, *n* = 48 days, for *q* = 1/2, and *n* = 192 days, for *q* = 1/8. The number of constructed windows for the upstream subsector case was *m* = 218 (when *q* = 1/2) and *m* = 176 (when *q* = 1/8). For the midstream subsector case *m* = 260 (for *q* = 1/2) and *m* = 235 (for *q* = 1/8). For the downstream case, *m* = 231 (for *q* = 1/2) and *m* = 222 (for *q* = 1/8). For all subsectors a skip of Δ*n* = 20 days of transactions has been considered for sucessive windows; e.g., windows are shifted successively 20 days.

### 4.2 Results and discussion

**Fig 1:**
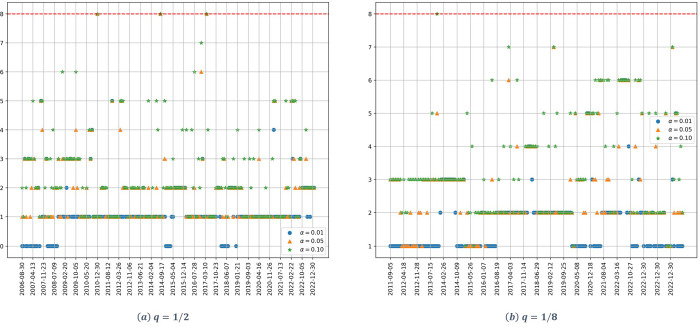
Number of factors as a function of time for the upstream stocks. (a) *q* = 1/2, (b) *q* = 1/8. The parameter *α* denotes the significance level of the Onatski test, and the red dotted line represents the alternative hypothesis’s upper bound *k*_2_.

Fig 1 shows the dynamic of the estimated number of significant factors for the upstream dataset.

Starting with the high dimensionality case (Fig 1A) and focusing on the confidence level *α* = 0.01, represented by the blue dots, just one significant factor is identified, with the exception of a small period, that goes from 2006 to the middle of 2007, for which no factors are identified. If we increase the level of confidence up to *α* = 0.05, just one significant factor is identified for the whole period, with the exception of two small periods, for which a two-factor model can be accepted.

For the case of low dimensionality (Fig 1B), considering a confidence level *α* = 0.01, just one significant factor is identified for the whole studied period. If we raise the confidence level up to 5%, again just one significant factor is identified, with the exception of two periods: from 2009 to mid-2010 and from 2018 to 2020; for which a 4 and 2 factors model can be accepted, respectively.

**Fig 2:**
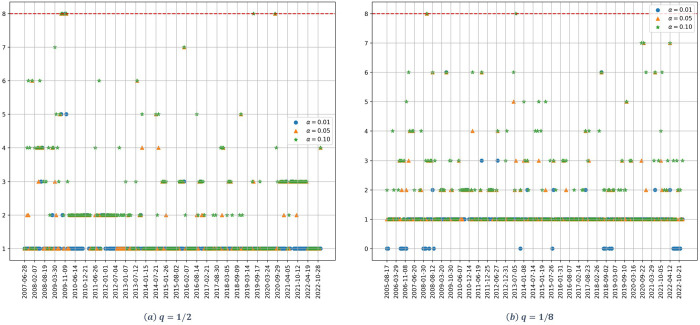
Number of factors as a function of time for the midstream stocks. (a) *q* = 1/2, (b) *q* = 1/8. The parameter *α* denotes the significance level of the Onatski test, and the red dotted line represents the alternative hypothesis’s upper bound *k*_2_.

Fig 2 shows the dynamic of the estimated number of significant factors for the midstream stocks. Starting with the high dimensionality case (Fig 2A), and with the exception of small time periods, for the three confidence levels there exists clearly just one significant factor.

For the low dimensionality case, when considering an *α* = 0.01, there is just one significant factor for the whole study period. For higher values of alpha, we accept the hypothesis of the existence of a two-factor model for the period from the end of 2009 until mid-2012.

**Fig 3:**
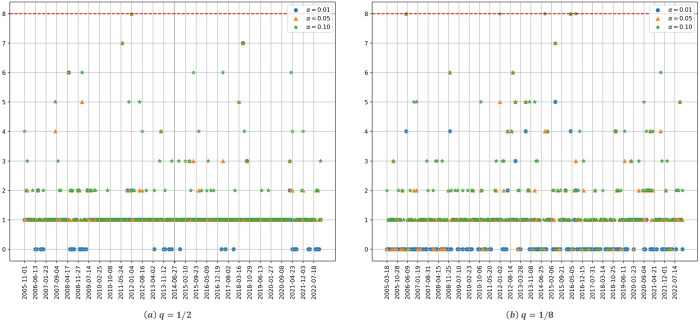
Number of factors as a function of time for the downstream stocks. (a) *q* = 1/2, (b) *q* = 1/8. The parameter *α* denotes the significance level of the Onatski test, and the red dotted line represents the alternative hypothesis’s upper bound *k*_2_.

Fig 3 shows the dynamic of the estimated number of significant factors for the downstream stocks. Starting with the case of high dimensionality (Fig 3A), and considering a confidence level *α* = 0.01, no statistically significant factors are identified for the studied period. Considering higher confidence levels (*α* = 0.05,0.10) just one significant factor is identified by the Onatski test. For the case of low dimensionality (Fig 3B), with the exception of small periods of time, one significant factor is identified for the three confidence levels employed in the experiment.

We wanted to go one step further and, as done in Molero-Gonzalez et al. [11], we wanted to proof that the identified factor is the market one. To do so, we have again done the experiment, trying to identify the number of significant factors, but now on the residual covariance matrix of the Sharpe’s Single Index model: $R_{it}=\alpha_{it}+\beta_{it}R_{mt}+\epsilon_{it},$where *R*_*it*_ is the return of asset *i* at time *t*, *R*_*mt*_ is the market return at time *t*, *α*_*it*_ is the minimum expected return of asset *i* at time *t*, *β*_*it*_ is the asset’s sensitivity to market’s movement, and ∈_*it*_ is the residual of the model at time *t*. In other words, what we do is to repeat the test again but now the data matrix *X* is not the covariance matrix of returns, but rather the covariance matrix of the residuals. By doing this what we do is to not consider the market, remove the market.

**Fig 4:**
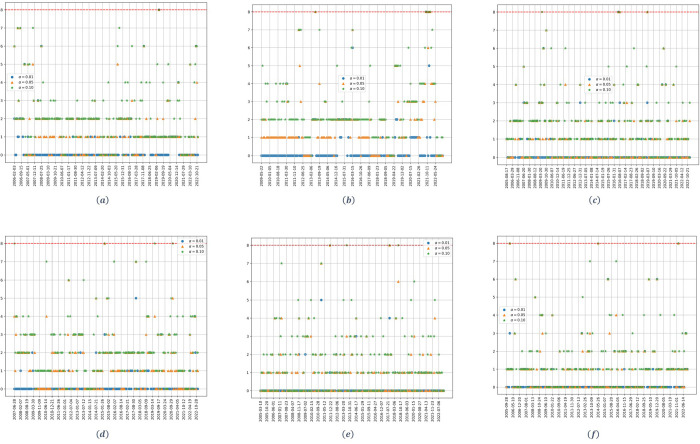
Number of factors as a function of time for the residual covariance matrix of the Sharpe model. (a) upstream at *q* = 1/2. (b) upstream at *q* = 1/8. (c) midstream at *q* = 1/2. (d) midstream at *q* = 1/8. (e) downstream at *q* = 1/2. (f) downstream at *q* = 1/8. The parameter *α* denotes the significance level of the Onatski test, and the red dotted line represents the alternative hypothesis’s upper bound *k*_2_.

Fig 4 shows the dynamic of the estimated number of significant factors when the market one is removed. As it can be seen, for the upstream stocks (Fig 4A and 4B), and for the three confidence levels, one less factor is identified, consistently, compared to the results obtained when applying the test to the complete model (Fig 1). This happens for both the high and the low dimensionality cases.

For the midstream case (Fig 4C and 4D) the results are almost the same for the high and the low dimensionality case. In both scenarios, when the market factor is removed, one less significant factor is identified for the whole period; so that, as it can be seen, no significant factors are identified for an *α* = 0.01.

For the downstream stocks (Fig 4E and 4F) the results obtained are quite similar. Starting with the high dimensionality case, remember that for the complete model (Fig 3) no significant factors were obtained for *α* = 0.01. For higher values of *α*, one factor was identified. When removing the market (Fig 4C), one less factor is identified by the Onatski test for the whole study period, meaning that the identified factor in Fig 3 is the market, but the market factor is less significant for these stocks. For the case of low dimensionality, the results follow those obtained for the upstream stocks.

A final step in this study has been to test whether this market factor identified as the only statistically significant factor could be the Brent crude oil price. To achieve this goal, we have repeated the experiment for the complete Sharpe model, taking the Brent crude oil closing prices as the market index. We have then again removed the market to test if the Brent crude oil price can be considered to be the only significant factor identified by the Onatski test.

**Fig 5:**
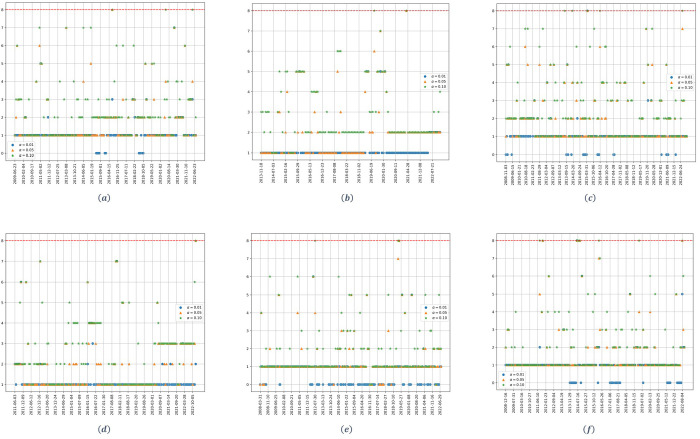
Number of factors as a function of time. (a) upstream at *q* = 1/2. (b) upstream at *q* = 1/8. (c) midstream at *q* = 1/2, (d) midstream at *q* = 1/8. (e) downstream at *q* = 1/2. (f) downstream at *q* = 1/8. The parameter *α* denotes the significance level of the Onatski test, and the red dotted line represents the alternative hypothesis’s upper bound *k*_2_.

Fig 5 shows the dynamic of the estimated number of significant factors, when the Brent crude oil price is taken as the market index, instead of taking the weighted market. Results obtained are completely the same as the ones showed in Figs 1–3.

**Fig 6:**
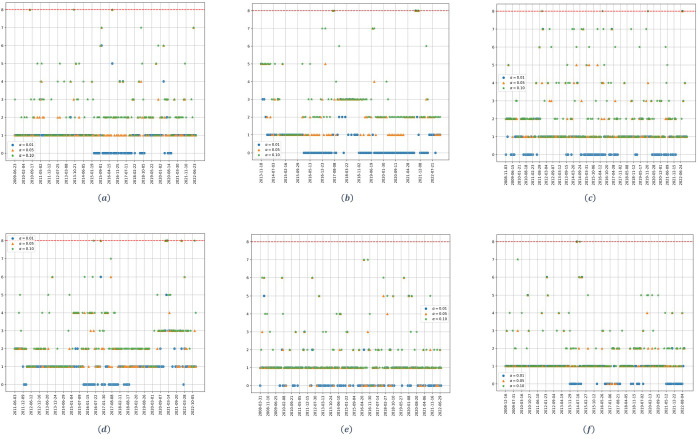
Number of factors as a function of time for the residuals covariance matrix of the Sharpe model. (a) upstream at *q* = 1/2. (b) upstream at *q* = 1/8. (c) midstream at *q* = 1/2. (d) midstream at *q* = 1/8. (e) downstream at *q* = 1/2. (f) downstream at *q* = 1/8. The parameter *α* denotes the significance level of the Onatski test, and the red dotted line represents the alternative hypothesis’s upper bound *k*_2_.

Fig 6 shows the dynamic of the estimated number of factors when the market factor (e.g., the Brent crude oil price) is removed. Starting with the upstream stocks (Fig 6A and 6B), and considering an *α* = 0.01, we can see that, for the high dimensionality case, one less significant factor is identified in comparison to Fig 5(A) for two periods of time: first, from 2015 to the beginning of 2018; and, second, in mid-2020. For the case of low dimensionality, the results are exactly the same, but the periods for which one less significant factor is identified are: from 2015 to the end of 2017 and from the end of 2018 to the beginning of 2020. For both cases, there are two periods in which the Brent crude oil price becomes the sole explanatory factor for the performance of the upstream stocks.

The results are quite similar for midstream stocks (Fig 6C and 6D). Starting with the high dimensionality case, and considering an *α* = 0.01, one less factor is identified for periods: 2010 and 2011, from the end of 2014 to the end of 2016; and from mid-2020 to the beginning of 2022. For the low dimensionality case, there is just one period, from the end of 2015 to the beginning of 2018, for which the market factor disappears.

The results obtained for the downstream stocks (Fig 6C and 6D) are very different. In this case, no fewer significant factors are identified for any of the studied scenarios; meaning that the Brent oil crude price never becomes an explanatory factor for the downstream stocks.

The behavior of the Brent oil price has a different impact on each subsector due to the nature of the activities involved in each one. In the Upstream subsector, companies are in charge of the exploration, extraction and exploitation of crude oil, selling it to Downstream companies for its subsequent refining, transportation and commercialization. Upstream companies have a direct and closer relationship with crude oil, and therefore with its reference price. This close relationship means that in our analysis we note that the Brent oil price is significant for the Upstream and Midstream subsector. The latter subsector is closely related to the Upstream. From a general standpoint, Midstream companies are in charge of the transportation of crude oil, logistic activities related to extraction, as well as drilling and management of oil wells. Authors such as Carson [54] warn that Exploration & Production and Equipment & Services companies (Upstream subsector) tend to have greater variation to market and commodity risk. Being closer to the physical recovery of oil and gas, these companies are more affected to price variation. On the other hand, as shown in the results, the Brent oil price is not a significant factor for companies included in the Downstream. The companies tend to have more bargaining power, passing on a rise in the crude oil price to the end consumer in the form of a rise in fossil fuels and oil products. Thus, when the Brent price rises, the operating cost of these companies is not affected, because it is balanced by the increase in their revenues caused by the increase in the retail price of their products and/or services [57].

In the results it can also be observed how the significance of the Brent oil price varies as a function of time. It is observed that the periods of significance are 2015–2018 and mid-2020. The significance is due to the high volatility that the Brent oil price experiences in these two periods. Between 2014 and 2016, the benchmark price decreases dramatically from $105.2 to $33.6. More recently, the COVID-19 pandemic produces another plummeting Brent oil price, causing between December 2019 and April 2020, the price to drop by 67% [82]. These two periods of high volatility could be observed in the previous analysis for the Upstream and Midstream subsectors.

As advanced in the Literature Review section, previous authors have studied the factors affecting stock performance in the O&G industry. In line with the results obtained, Adekunle et al. [30] warns that oil price affects the prediction of earnings per share in a set of O&G companies in Nigeria. Others such as Sanusi and Ahmad [9] obtain similar results for the O&G industry in United Kingdom. The authors show that variables such as price risk, company size and market risk can influence stock performance. In our case, the market factor is also significant in the three subsectors of study, going in line with previous literature and demonstrating that the behavior found in specific geographic markets could be extrapolated to a global sample.

Seminal articles such as Sadorsky [18] demonstrate for the Canadian O&G industry that the market, the price of crude oil, the exchange rate and interest rates are explanatory factors for the behavior of the TSE Oil and Gas Index. In line with these results, more recent authors such as Gupta [83] and Hoque and Low [34] have verified that there are macroeconomic variables that can affect on the behavior of the O&G industry. In the present study, it is observed how in certain periods a model of up to 4 factors can be accepted. Further analysis may conclude that these factors are macroeconomic as indicated by previous literature.

Periods of high volatility and financial distress may lead companies to consider a cost-cutting strategy, thus reducing revenues, the profitability, and ultimately negatively affecting the share price. This is what authors such as Osmundsen et al. [84, 85] found for example. Despite this relationship proven by previous literature, a significant relationship has not always been found. Mohanty et al. [52] argue that there is no significant link between oil price and stock returns for a set of O&G companies in Central and Eastern European (CEE) countries. However, the authors find that in periods of financial crisis (2008–2009), significant systematic risk is attributed to stock return performance. These results are in line with the conclusions obtained in this article, as it has been found that for certain periods and subsectors, the Brent oil price is not significant. From the wavelet analysis, Reboredo and Rivera-Castro [25] warn that changes in oil prices have no effect on stock prices in pre-crisis periods, with the exception of O&G stocks. For Liu [7], short-term investors need not be affected by oil price risk when investing in O&G companies, but long-term investors should consider this issue. This price behavior suggests that investors and shareholders should consider when to invest in these companies. Due to their high volatility, and the possible macroeconomic factors that affect the share price, it is crucial to know the factors that explain the movement in the shares, especially when we are in periods of financial crisis and oil price volatility. A differentiation by subsectors is also crucial to understand the specific behavior of each company, due to the nature of its activity. This perspective can make the decisions taken more correct according to the type of company within the O&G chain.

## 5. Conclusions

For several decades, the literature has been trying to explain which factors affect the O&G stock returns. Much of the literature analyzed indicates that there are significant and relevant factors affecting these companies, such as the Brent benchmark price [9], the interest rate [48], the exchange rate [35] or the four factors of the Fama French-Carhart’s factor model [29]. However, authors such as Chang et al. [51] did not find significant spillover effects between oil and stock returns for the period 1996–2009. Others such as Mohanty et al. [52] are in line with this result for a sample of Central and Eastern European (CEE) countries for the period 1998–2010. These mix results generate a debate that continues to this day and, considering the high relevance of the sector, it is a priority to contribute to clarify this issue. This paper proposes to continue the relentless search for explanatory factors from a novel approach based on Random Matrix Theory (RMT) models. A purely statistical methodology that allows us to test the robustness of the multifactor models used to explain the movements in the O&G sector (2020).

After performing the analysis across the different subsectors that make up the O&G sector chain, it can be seen that for O&G companies, only one factor has been identified as statistically significant in explaining the O&G stock returns. This factor is the market. For upstream and midstream companies, the market is significant at a 1% alpha confidence level. For downstream companies, the market is less significant (only significant at higher alpha levels of 5% and 10%). For upstream and midstream companies, the closing price of the Brent becomes the only explanatory factor for certain periods. However, for downstream companies, Brent is never an explanatory factor of the cross-sectional expected return. The results obtained allow to rethink the analysis made by investors, executives and politicians when making decisions concerning a highly relevant sector such as the O&G.

While it is true that this study uses a methodological approach that differentiates it from the rest of the literature analyzed, it is not without limitations. A geographical differentiation could give more reliable results in order to check whether the O&G market is influenced by specific characteristics of each region [83]. Another issue to consider is the non-appearance of explanatory factors in the downstream subsector at 1% level. A specific focus on the different activities involved in the downstream subsector or a sample divided by regions could give greater depth to the possible significant factors, considering levels of 5% or 10%.

## Supporting information

S1 Table Critical values for the hypothesis test. The rows represent the level of significance and the columns the size of the test. Source: Onatski (2009).
(DOCX)

S1 Code (DOCX)
