# Supplementary material for: Testing the significance of pricing factors of oil and gas companies
Source: PLoS One. 2024 Dec 30;19(12):e0316147. doi: 10.1371/journal.pone.0316147 (PMC11684706; doi:10.1371/journal.pone.0316147)
Supplement: S1 Table — The rows represent the level of significance and the columns the size of the test. Source: Onatski (2009). (DOCX) [file pone.0316147.s001.docx]

**Table S1. Critical values for the hypothesis test**. The rows represent the level of significance and the columns the size of the test.

| Size  % | *k*_1_ − *k*_0_ | | | | | | | |
| --- | --- | --- | --- | --- | --- | --- | --- | --- |
|  | 1 | 2 | 3 | 4 | 5 | 6 | 7 | 8 |
| 15 | 2.75 | 3.62 | 4.15 | 4.54 | 4.89 | 5.2 | 5.45 | 5.7 |
| 10 | 3.33 | 4.31 | 4.91 | 5.4 | 5.77 | 6.13 | 6.42 | 6.66 |
| 9 | 3.5 | 4.49 | 5.13 | 5.62 | 6.03 | 6.39 | 6.67 | 6.92 |
| 8 | 3.69 | 4.72 | 5.37 | 5.91 | 6.31 | 6.68 | 6.95 | 7.25 |
| 7 | 3.92 | 4.99 | 5.66 | 6.24 | 6.62 | 7 | 7.32 | 7.59 |
| 6 | 4.2 | 5.31 | 6.03 | 6.57 | 7 | 7.41 | 7.74 | 8.04 |
| 5 | 4.52 | 5.73 | 6.46 | 7.01 | 7.5 | 7.95 | 8.29 | 8.59 |
| 4 | 5.02 | 6.26 | 6.97 | 7.63 | 8.16 | 8.61 | 9.06 | 9.36 |
| 3 | 5.62 | 6.91 | 7.79 | 8.48 | 9.06 | 9.64 | 10.11 | 10.44 |
| 2 | 6.55 | 8.15 | 9.06 | 9.93 | 10.47 | 11.27 | 11.75 | 12.13 |
| 1 | 8.74 | 10.52 | 11.67 | 12.56 | 13.42 | 14.26 | 14.88 | 15.25 |

Source: Onatski (2009)
