# Supplementary material for: Testing the significance of pricing factors of oil and gas companies
Source: PLoS One. 2024 Dec 30;19(12):e0316147. doi: 10.1371/journal.pone.0316147 (PMC11684706; doi:10.1371/journal.pone.0316147)
Supplement: S1 Code — (DOCX) [file pone.0316147.s002.docx]

**Code availability**

Random Matrix Theory’s codes employed on this research are available at:

<https://github.com/agarciam/RMT_statistical_tests/>
